# Effects of biochar amendment and organic fertilizer on microbial communities in the rhizosphere soil of wheat in Yellow River Delta saline-alkaline soil

**DOI:** 10.3389/fmicb.2023.1250453

**Published:** 2023-09-22

**Authors:** Meng Li, Chuanjie Chen, Haiyang Zhang, Zongshuai Wang, Ningning Song, Junlin Li, Xiaoyan Liang, Kuihua Yi, Yinyu Gu, Xiaohong Guo

**Affiliations:** ^1^Shandong Institute of Sericulture, Shandong Academy of Agricultural Sciences, Yantai, China; ^2^Crop Research Institute, Shandong Academy of Agricultural Sciences, Jinan, China; ^3^School of Resources and Environment, Qingdao Agricultural University, Qingdao, China; ^4^School of Resources and Environmental Engineering, Ludong University, Yantai, China

**Keywords:** biochar, organic fertilizer, microbial communities, wheat, saline-alkali soil

## Abstract

The biochar and organic fertilizer amendment have been used as an effective practice to increase soil fertility. Nevertheless, the mechanisms of microbial community response to organic fertilizer and biochar application on saline-alkali soil have not been clarified. This study investigated the effects at different concentrations of organic fertilizer and biochar on the microbial community of wheat rhizosphere soil under field experiment in the Yellow River Delta (China, YRD), using high-throughput sequencing technology. Biochar and organic fertilizer significantly influenced in most soil parameters (*p* < 0.05), apart from soil moisture content (M), pH, total nitrogen (TN) and soil total phosphorus (TP). Proteobacteria and Actinobacteriota were found in the rhizosphere soil as the main bacterial phyla, and the main fungal phyla were Ascomycota and Mortierellomycota. The soil bacterial and fungal communities under organic fertilizer were distinct from CK. Furthermore, redundancy analysis (RDA) directed that changes in bacterial communities were related to soil properties like pH, available phosphorus (AP), and total organic carbon (TOC), while pH, AP and TP, were crucial contributors in regulating fungal distribution. The correlation between soil parameters and bacteria or fungi varied with the application of biochar and organic fertilizers, and the interaction between the bacteria and fungi in organic fertilizer treatments formed more connections compared with biochar treatments. Our results indicated that biochar was superior to organic fertilizer under the contents set up in this study, and soil parameters increased with biochar and organic fertilizer application rate. The diversity and structure of soil bacteria and fungi differed with the application of biochar and organic fertilizer. The research provides a reference to rational application of organic fertilizer and biochar improvement in saline-alkali soil.

## 1. Introduction

The Yellow River Delta (YRD), located in the warm-temperate zone, is gradually being degraded as one of the important deltaic agricultural economic zones in China (Fang et al., 2005; Luo et al., 2016; You et al., 2021). The saline-alkali land of the YRD is approximately 2,400 km^2^, which is about half of the area. Restricted by salinity stress and nutrient deficiencies, the vegetation structure in this area is simple and biodiversity is low (Luo et al., 2016; Li et al., 2019). Soil microorganisms play an important controller for plant growth and stress tolerance and are often used as an indicator of soil quality within evaluations (Salas-González et al., 2020; Wang et al., 2021). Consequently, appropriate amendments need to be selected which can improve the soil physicochemical and biological properties in order to realize sustainable usage of saline-alkali soil in the YRD.

Organic fertilizers are a positive option for regulating soil properties and plant growth in degraded soil (Le et al., 2016). Application of organic fertilizers in saline-alkali soil offers a better option for increasing organic carbon content in soil and crop yields by providing essential plant nutrients and organic materials (Demelash et al., 2014). Many searches have indicated that the addition of organic fertilizers to soil enhances organic carbon content, soil structure, cation-exchange capacity, and nutrient quality (Le et al., 2016; Gu et al., 2023). Organic fertilizers are also known to stimulate soil microbial biomass (Zhao et al., 2020; Liu et al., 2021), enzyme activities (Plaza et al., 2004; Ge et al., 2009; Insam et al., 2015), and to promote changes in community structure and abundance (Zhao et al., 2016). Nevertheless, organic fertilizers application does not always enhance soil microbial diversity, their impact depends on the duration of application, the source and nature of the fertilizer, soil type, and tillage conditions (Xu et al., 2021). Thus, the impacts of different quantities of organic fertilizer on the soil microbial community should be investigated using organic fertilizers with the goal of reclaiming saline-alkali soils in the YRD.

Biochar has attracted tremendous attention for its soil ameliorating effects, and it enhances carbon storage, soil fertility and quality, and contaminant immobilization and transformation (Zhu et al., 2017). Biochar is an alkaline material, rich in recalcitrant carbon and surface functional groups (Lehmann et al., 2011; Zhou et al., 2019). Because of these reasons, biochar has also been considered as a popular and promising soil conditioner that can improve crop yields (Kang et al., 2018; Zhang et al., 2019). In most researches, biochar has been able to increase the biomass of microorganisms in the soil, with significant changes in microbial community composition possibly explain the biogeochemical impacts of biochar on element cycles, pathogenic bacteria, and crop growth (Lehmann et al., 2011). Several studies have demonstrated that biochar indeed affects soil microbial communities (Chen et al., 2013; Liu et al., 2017; Zhang et al., 2018), and have reported possible effects of biochar on microbial community abundance and structure (Sun et al., 2013). These conflicting results are mainly due to variations in soil type, biochar variety, biochar production conditions and duration, application rate, and time (Dangi et al., 2019). Thus, understanding the soil microbial community and its reactions to diverse biochar amendments will inform a new approach for alleviating poor soil physicochemical and biological properties, and thus provide a practical management approach for sustainable agricultural production in saline-alkali soils.

Wheat (*Triticum aestivum*) is a leading staple crop and is cultivated all over the world, with approximately 2.1 million km^2^ under cultivation (Meng et al., 2019). Previous study observed that the use of organic fertilizer and biochar could improve soil quality and performance of wheat (Meng et al., 2019). Researches have also indicated that rhizosphere bacteria and fungi can contribute directly to plant growth (Compant et al., 2010; Lehmann et al., 2011). However, in saline-alkali soil, the reaction of the rhizosphere microbial community after organic fertilizer and biochar amendment applications has not been well investigated. Therefore, in this study, the YRD saline-alkali soil was used as the research target to determine the effects of different amounts of organic fertilizer and biochar additions on (i) the nutrient profile and physicochemical characteristics of the soil; and (ii) the rhizosphere bacterial and fungal communities of wheat in the target site. We hypothesized that moderate amounts of organic fertilizer and biochar amendment would enhance soil physicochemical properties and create soil bacterial and fungal communities which alleviate stress induced by saline-alkali soil in the YRD.

## 2. Materials and methods

### 2.1. Biochar and organic fertilizer

The biochar used in this study was sourced from Taiyu Bioengineering Co., Ltd. (Qixia, China). It was derived from apple shoots and processed at a temperature of 450°C for 1 day. The biochar had a pH value of 7.52 and an electrical conductivity (EC) of 0.35 ms/cm. And it contained 70.2% carbon, 0.35% nitrogen, 0.13% available phosphorus, and 1.53% available potassium. Organic fertilizer was provided by Yangfeng Agricultural Technology Co., Ltd. (Weifang, China), based on maize straw and mushroom residue as the main composting substrate. The pH value of organic fertilizer was 7.94, with an EC of 3.25 ms/cm. The organic fertilizer had a high organic matter content more than 60% and nitrogen, phosphorus, and potassium contents more than 6%.

### 2.2. Field experiments

Field experiments were made in the Yellow River Delta Institute of Modern Agriculture, Shandong Academy of Agricultural Sciences, Dongying, China (118.37°N, 37.17°E). The primary soil type of the experimental plot was a typical saline-alkali of YRD, the specific texture and type of which are shown in Gu et al. (2023). Winter wheat (*Triticum aestivum* L.) was sown in October 2017and harvested in June 2018.We established six treatments with three replications: no biochar or organic fertilizer (CK); low biochar (BL): 10.0 t/ha; medium biochar (BM): 20.0 t/ha; high biochar (BH): 30.0 t/ha; low organic fertilizer (ML): 7.5 t/ha; medium organic fertilizer (MM): 10.0 t/ha. Test plot was designed in random block design with three replicates, the plot area was 10 m × 15 m with 1 m gaps between plots. Organic fertilizer and biochar were spread on the soil surface and then evenly tilled 0–20 cm before planting the crop. Other field management followed local management practice. At the end of the field trial, wheat rhizosphere soil was obtained from the tilled area (0–20 cm) and collected and processed according to Gu et al. (2022a). Then, the collected samples were split into two parts: one for high-throughput sequencing, the other for soil physicochemical properties after being air-dried to remove impurities.

### 2.3. Analysis of soil physicochemical properties

Soil moisture content (M) was measured by the weight method. pH was measured with a water quality analyzer (Shanghai Lechen LC-MP-41 T). Soil electrical conductivity (EC) was measured at 10 cm soil level with a conductivity meter (SANXIN SX836). Other nutrients were assayed according to our previous study (Gu et al., 2023). Alkaline nitrogen (AN) and total nitrogen (TN) contents of soil were analyzed using the Analytik Jena Nitrogen Elemental Analyzer Multi-N/C 2100/2100S, Germany. Available phosphorus (AP) was extracted by NaHCO_3_ and measured on a continuous flow analyzer (AMS Alliance, Futura, France). Soil total phosphorus (TP) was measured by wet digestion with HClO_4_-H_2_SO_4_ and quantified using inductively coupled plasma emission spectrometry (Agilent 5,800 ICP-OES, United States). Available potassium (AK) was with CH_3_COONH_4_ (pH 7.0) and measured by inductively coupled plasma emission spectrometer (Agilent 5,900 SVDV, USA). Organic matter in soil was quantified using a potassium dichromate oxidation and carbon analyzer (OI Analytical Aurora 1,030 TOC, USA).

### 2.4. Soil microbial community analysis

DNA was extracted from wheat rhizosphere soil samples by FastDNA® Spin Kit for Soil (MP Biomedicals, USA) and the quality of the extraction was checked by NanoDrop 2000 luminometer. The extracted DNA was amplified by polymerase chain reaction (PCR) technique. The 16S gene in the V3-V4 region of the rhizosphere soil samples was sequenced using primers 338F (5′-ACTCCTACGGGAGGCAGCAG-3′) and 806R (5′-GGACTACHVGGGTATCTAAT-3′), while the internal transcribed spacer (ITS) were sequenced using primers ITS1F (5′-CTTGGTCATTTAGAGGAAGTAA-3′) and ITS2R (5′-GCTGCGTTCTTCATCGATGC-3′). All sequencing was performed in triplicate and the results obtained are stored in the NCBI SRA database (PRJNA987399).

### 2.5. Data analysis

All data were calculated using Excel 2023 for mean and standard deviation. Duncan multiple range test and Pearson correlation analysis were conducted with DPS Statistics 18.10. Data related to microorganisms are counted and processed on the Majorbio Cloud Platform^1^ Figures were performed using Origin Pro 2022 software.

## 3. Results

### 3.1. Soil physicochemical properties

The impact of organic fertilizer and biochar application on the soil properties and nutrient profile of saline-alkali soil is shown in Table 1. In general, biochar amendment and organic fertilizer caused significant changes in most soil parameters (*p* < 0.05), apart from M, pH, TN and TP. Compared to CK, all treatments significantly reduced the EC and increased AK (*p* < 0.05). The concentration of AN was significantly increased in BH as compared to CK and the BL treatment (*p* < 0.05), while being no significantly different from the other treatments. In comparison to CK, AP levels were significantly increased under BL, BM, and BH treatments (*p* < 0.05), however, the AP levels were also significantly decreased by the ML and MM treatments compared with CK (*p* < 0.05), but the increase was lower than the BL, BM, and BH treatments. The level of TOC was significantly higher in the BL, BM, and BH treatments than in CK (*p* < 0.05), while in the ML and MM treatments compared to CK no obvious changes were observed. The TC level was significantly higher in the BL, BM, BH and MM treatments as compared to CK and the ML treatment (*p* < 0.05).

**Table 1:** Effect of biochar and organic fertilizer on the physical and chemical properties of soil.

	CK	BL	BM	BH	ML	MM
M(%)	7.72 ± 1.00a	8.36 ± 1.71a	7.92 ± 1.82a	8.22 ± 0.94a	8.88 ± 2.51a	9.64 ± 3.17a
pH	8.04 ± 0.087a	8.05 ± 0.032a	8.07 ± 0.042a	8.06 ± 0.081a	7.99 ± 0.036a	7.98 ± 0.040a
EC(ms/cm)	370.89 ± 16.45a	309.33 ± 19.36d	266.76 ± 17.81e	332.78 ± 19.71b	319.77 ± 13.61c	333.33 ± 18.57b
AN(mg/kg)	100.93 ± 7.57b	97.60 ± 9.32b	107.48 ± 7.40ab	118.03 ± 3.22a	104.94 ± 4.16ab	103.43 ± 9.75ab
TN(g/kg)	1.49 ± 0.08a	1.53 ± 0.19a	1.62 ± 0.16a	1.57 ± 0.21a	1.44 ± 0.13a	1.58 ± 0.22a
AP(mg/kg)	59.17 ± 3.85d	80.01 ± 4.35c	87.82 ± 4.05b	95.48 ± 5.06a	41.26 ± 2.37f	51.18 ± 3.63e
TP(g/kg)	1.13 ± 0.06ab	1.27 ± 0.19a	1.29 ± 0.03a	1.30 ± 0.12a	1.06 ± 0.02b	1.08 ± 0.07b
AK(mg/kg)	195.29 ± 7.96d	211.21 ± 10.55c	235.37 ± 8.73b	273.10 ± 11.26a	205.40 ± 9.52c	241.18 ± 12.41b
TOC(g/kg)	9.48 ± 0.09d	13.83 ± 1.85c	18.53 ± 3.70b	21.92 ± 3.94a	9.90 ± 0.36d	10.85 ± 2.94 cd
TC(g/kg)	16.33 ± 1.15e	23.84 ± 1.19c	31.94 ± 1.73b	37.80 ± 1.80a	17.07 ± 1.62e	18.71 ± 1.06d

Data represent mean ± standard deviation (SD) of three biological replicates. Different lowercase letters within a row indicate significant differences (*p* < 0.05).

### 3.2. Microbial α-diversity

After read-quality filtering, 1,378,473 high-quality bacterial sequences and 1,017,877 high-quality fungal sequences were obtained. Bacterial and fungal sequences in this study were clustered into 2,326 and 309 OTUs, respectively, when grouped at 97% sequence similarity. Bacterial and fungal OTUs sparsity curves inclined towards a saturation plateau, which demonstrated that the data were large enough to detect most of the bacterial and fungal taxa in the rhizosphere soil (Supplementary Figure S1). As shown in Table 2, the OTUs and the Shannon and Chao1 index were compared under various conditions in the bacterial and fungal colonies, respectively. The OTUs and the Shannon and Chao1 index of the bacterial community amongst the six conditions were: 2109.33–2150.67 (OTUs), 6.31–6.50 (Shannon), and 2231.69–2253.98 (Chao1). For the fungal community, the values were: 192.33–242.00 (OTUs), 2.58–3.49 (Shannon), and 216.42–254.68 (Chao1; Table 2). There were no significant differences in OTU richness or the Chao1 index of the bacterial communities among the six conditions (*p* > 0.05). Nevertheless, the Shannon index of the bacterial communities was significantly increased in the BM treatment versus with the CK and MM treatment (*p* < 0.05), and was not significantly affected compared with others. When compared to CK, the OTUs or Shannon and Chao1 indices of the fungal communities under biochar amendment and organic fertilizer treated were no significant differences (*p* > 0.05).

**Table 2:** Operational taxonomic unit richness and diversity indices of different samples.

Treatment	Bacterial index			Fungal index		
	OTUs	Shannon	Chao 1	OTUs	Shannon	Chao 1
CK	2127.33 ± 38.81a	6.35 ± 0.10bc	2236.60 ± 34.49a	213.33 ± 12.50ab	3.23 ± 0.30a	238.34 ± 9.02a
BL	2129.33 ± 50.46a	6.46 ± 0.08ab	2235.80 ± 43.47a	229.00 ± 9.17ab	3.44 ± 0.50a	254.46 ± 14.35a
BM	2150.67 ± 48.34a	6.50 ± 0.09a	2249.28 ± 42.01a	227.33 ± 10.12ab	3.23 ± 0.36a	245.13 ± 8.27a
BH	2141.33 ± 36.23a	6.43 ± 0.08ab	2246.95 ± 24.44a	242.00 ± 10.82a	3.49 ± 0.30a	254.68 ± 13.61a
ML	2125.33 ± 45.76a	6.46 ± 0.06ab	2231.69 ± 31.98a	192.33 ± 33.50b	2.58 ± 0.83a	216.42 ± 44.35a
MM	2109.33 ± 36.53a	6.31 ± 0.02c	2253.98 ± 20.00a	208.67 ± 34.99ab	3.01 ± 0.69a	224.55 ± 32.68a

Data represent mean ± standard deviation (SD) of three biological replicates. Different lowercase letters within a row indicate significant differences (*p* < 0.05).

### 3.3. Microbial community composition and structure

**Figure 1:**
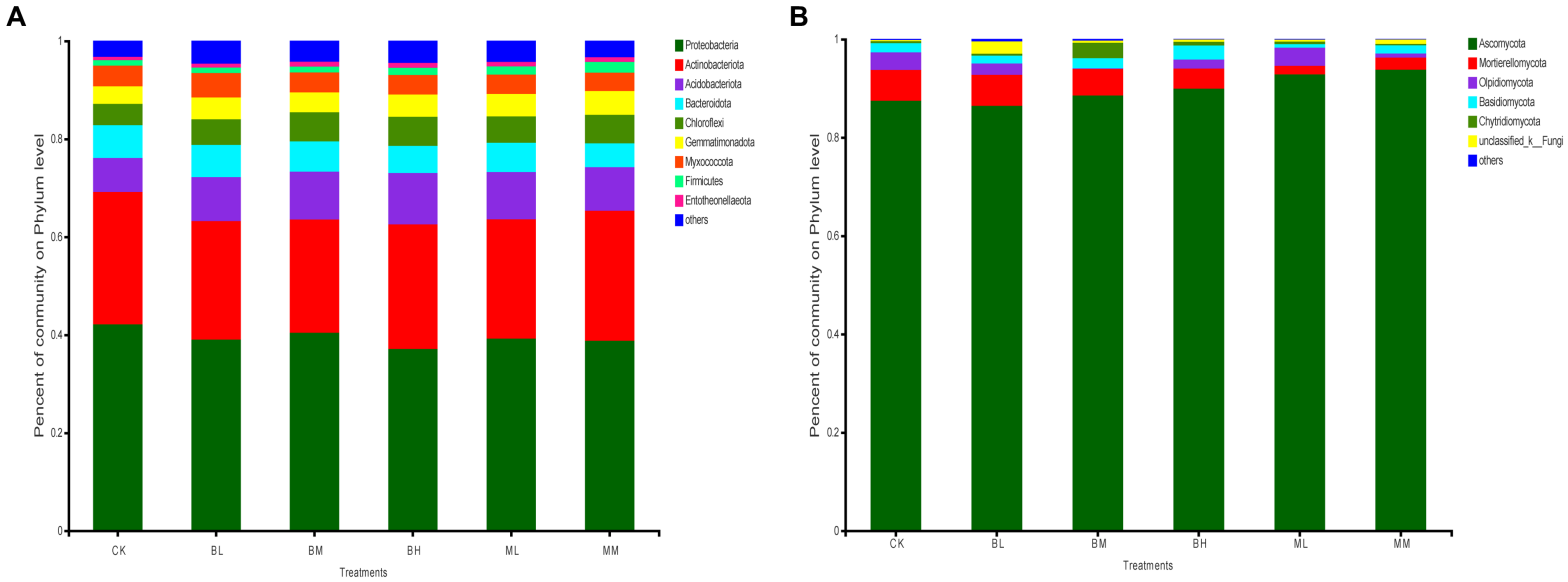
The relative abundances of bacteria **(A)** and fungi **(B)** at the phylum level in wheat rhizosphere soil.

After classification analysis, wheat rhizosphere soil was identified for 32 bacterial phyla and 11 fungal phyla. The most abundant bacterial phyla were Proteobacteria, Actinobacteriota, Acidobacteriota, Bacteroidota, and Chloroflexi across the soil samples, which represented more than 80% of the total bacterial population (Figure 1A). Of these, the highest relative abundance of Proteobacteria was found in all treated soils, which was followed by the Actinobacteria group. Ascomycota was the richest fungal community in the soil samples in terms of relative abundance, which ranged from 86 to 94% of the total communities tested (Figure 1B). Mortierellomycota ranked second for relative abundance in CK, BL, BM, BH, and MM, while Olpidiomycota ranked second for relative abundance in ML (Figure 1B).

**Figure 2:**
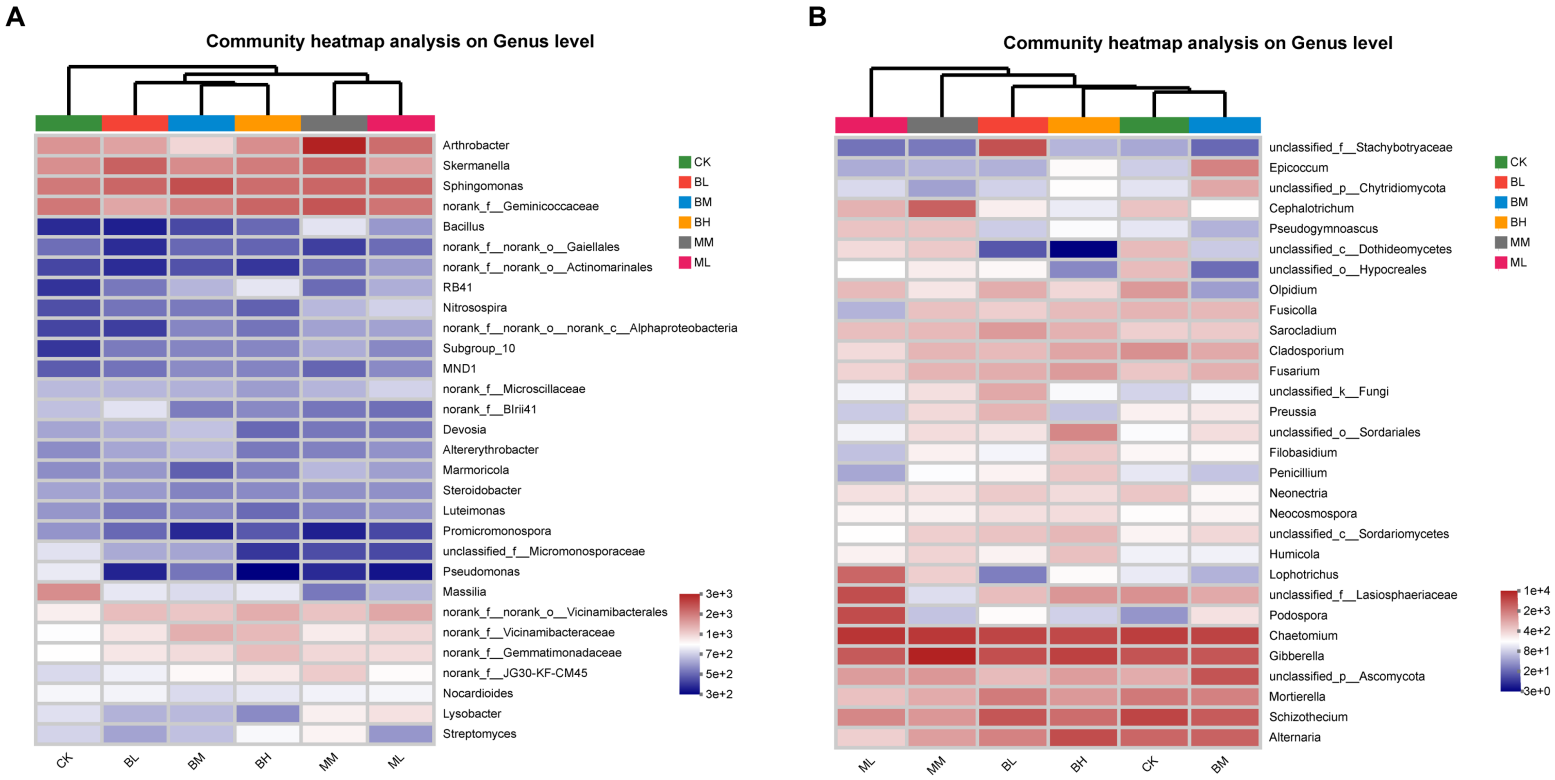
Heatmap of the top 30 classified genera of bacteria **(A)** and fungi **(B)** in wheat rhizosphere soil.

The heatmap of the 30 genera of most abundant classified bacterial and fungal were showed in Figure 2. At the bacterial genus level, *Sphingomonas*, *norank_f__Geminicoccaceae*, *Skermanella* and *Arthrobacter* were the top 4 dominant genera, and their relative abundances together represented approximately 13.4% of all sequences (Figure 2A). The distribution of the genera differed significantly across the different samples and belonged to eight phyla, including Proteobacteria (13 genera), Actinobacteriota (7), Acidobacteriota (4), Bacteroidota (1), Chloroflexi (1), Firmicutes (1), Gemmatimonadota (1), Myxococcota (1). Sphingomonas was the predominant bacterial genus in the BL, BM, and ML, while *norank_f__Geminicoccaceae* was the predominant bacterial genus in CK, BH, MM. *Skermanella* and *Arthrobacter* were the predominant bacterial genus in BL and MM, respectively. The heatmap of bacterial genera separated the samples from different treatments into three groups, with BL, BM and BH together and ML and MM together separated from CK. At the fungal genus level, *Chaetomium*, *Gibberella*, *Schizothecium*, *Alternaria* were the top 4 dominant genera, and their relative abundances together represented approximately 37.6% of all sequences (Figure 2B). The distribution of the genera differed significantly across the different samples and belonged to six phyla, including Ascomycota (25 genera), Basidiomycota (1), Chytridiomycota (1), Mortierellomycota (1), Olpidiomycota (1), unclassified_k__Fungi (1). *Chaetomium* was the predominant bacterial genus in the CK, BL, BM, and ML, *Gibberella* was the predominant bacterial genus in BH and MM. The heatmap of fungal genera separated the samples from different treatments into two groups, with CK, BL, BM and BH together separated from ML and MM together. These results suggest that the effects of organic fertilizer treatment on bacterial and fungal communities are different compared to CK and biochar treatment.

**Figure 3:**
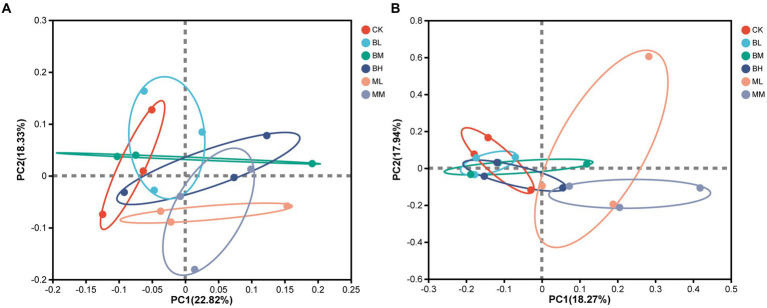
PCoA analysis of bacterial **(A)** and fungal **(B)** community structures based on Bray-Curtis distance at the OTU level.

A distinct distinction between soil bacterial and fungal communities under various treatments was made by principal coordinate analysis (PCoA) at the OTU level (Figures 3A,B). Coordinate axes 1 and 2 elucidate 22.82 and 18.33% for bacterial community variation and 18.27 and 17.94% for fungal community variation under different treatments, respectively. The results showed that the soil bacterial and fungal communities in BL, BM, and BH were similar to those in CK. However, the soil bacterial and fungal communities in ML and MM were distinct from those of CK along PCoA1 (Figure 3).

**Figure 4:**
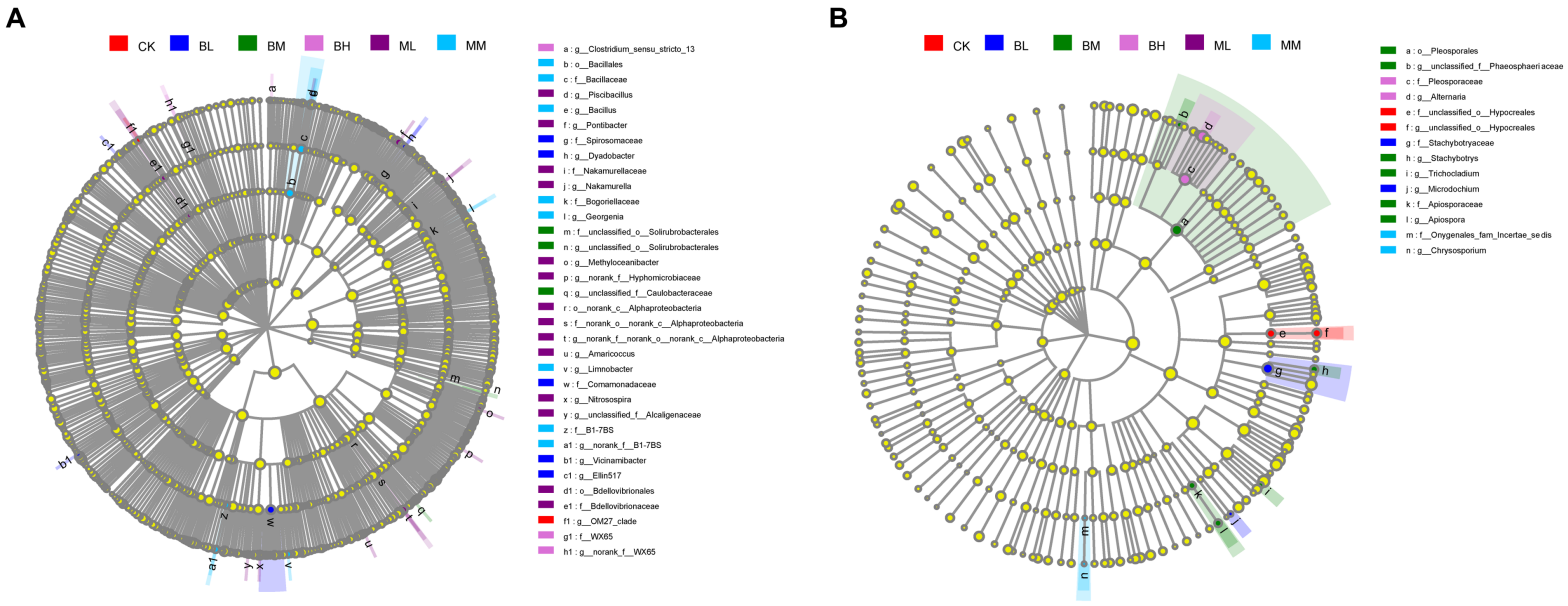
LEfSe analysis for **(A)** bacterial and **(B)** fungal community of treatments control.

Analysis by LEfSe showed that there were 35 bacterial and 14 fungal taxa enriched (α < 0.01, linear discriminant analysis (LDA) score > 2.0) in the six treatments (Figure 4). The bacterial and fungal taxa that were significantly enriched differed between treatments. The index microbes (LDA threshold of 2.0) in the microbial communities relevant to the treatment groups are shown in Supplementary Figures S2A,B, respectively. Of these, the largest number of bacterial species was enriched in the ML treatment with 9, followed by BL with the second largest at 7. The largest variety of fungal species enriched in the BM treatment was the largest at 6; no abundant fungal taxa were identified in the ML treatment.

**Figure 5:**
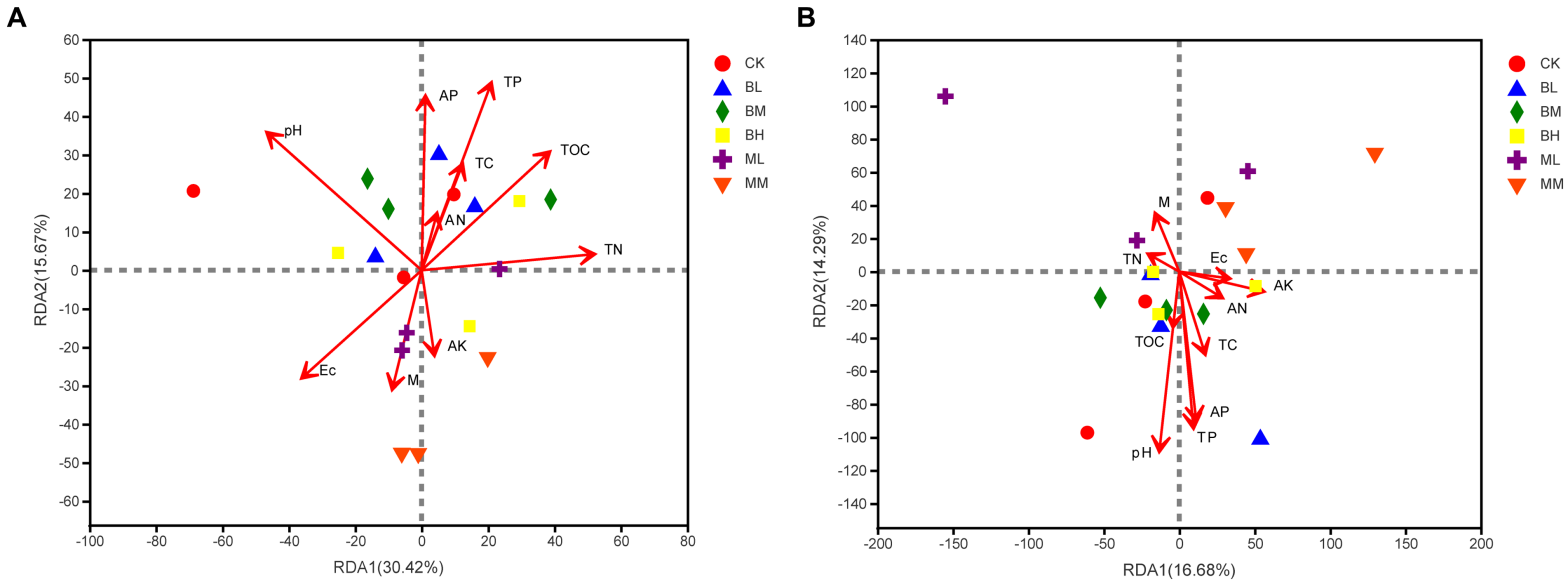
RDA of the relationships between soil bacterial **(A)** and fungal **(B)** community with soil properties.

The Redundancy analysis (RDA) was used to explain the impact of soil parameters on microbial populations at the genus level. These soil indicators elucidated 46.09% of the total variation in the soil bacterial community (RDA1, 30.42%; RDA2, 15.67%; see Figure 5A). It was identified that soil pH, TP, TN, and TOC were the main factors to cause changes in bacterial community variation (Supplementary Table S1). For the fungal communities, RDA1 and RDA2, respectively, explained 16.68 and 14.29% of the total variation (Figure 5B). Furthermore, soil pH, TP and AP were significant factors influencing the rhizosphere fungal community (Supplementary Table S2).

**Figure 6:**
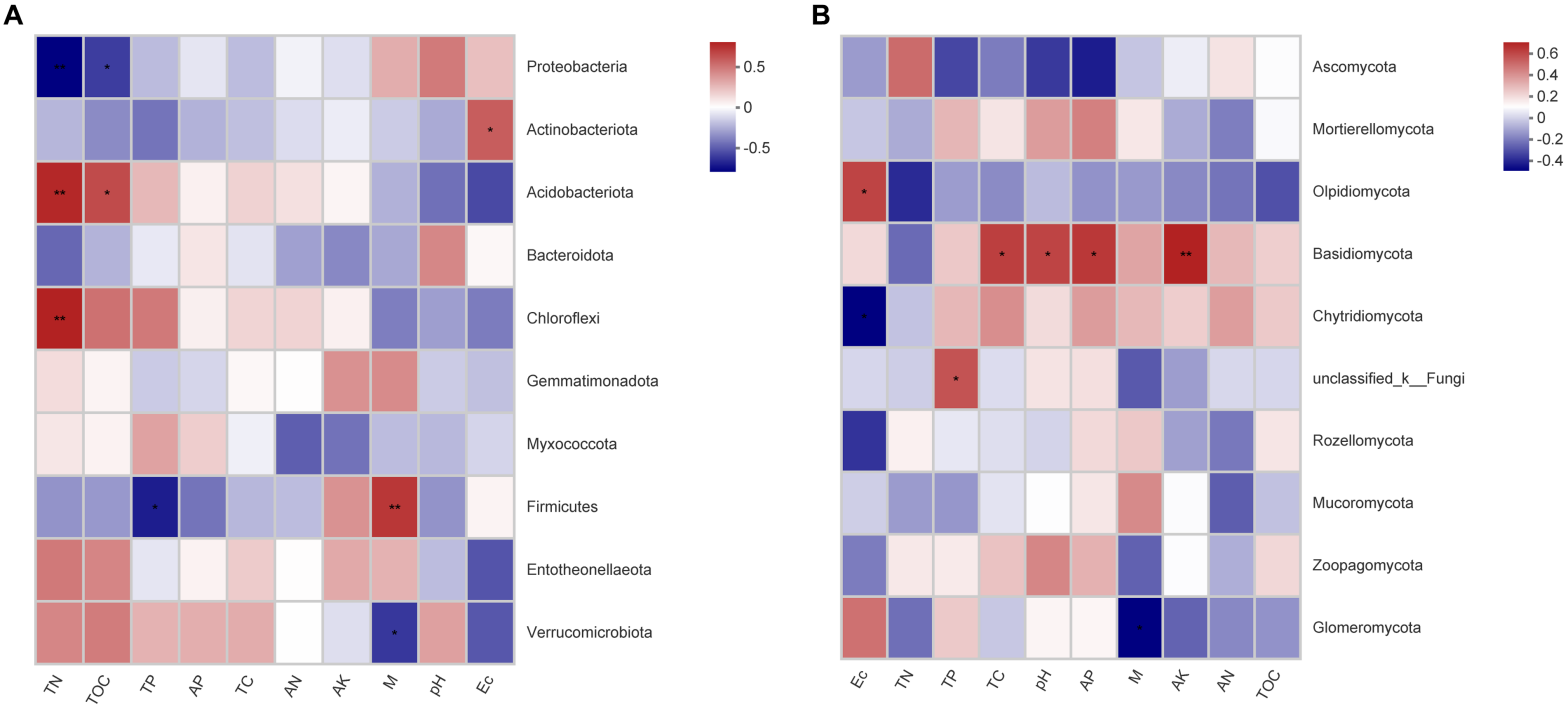
Heatmap of the Spearman correlation between the predominant **(A)** bacterial and **(B)** fungal phyla and environmental variables. *Correlation is significant at the 0.05 level (two-tailed). **Correlation is significant at the 0.01 level (two-tailed).

Correlation analysis between soil parameters and bacterial communities showed that Proteobacteria was significant negatively associated with TN (*p* < 0.01) and TOC (*p* < 0.05), while Actinobacteriota was significant positively associated with EC (*p* < 0.01), Acidobacteriota was significant positively associated with the TN (*p* < 0.01) and TOC (*p* < 0.05), Chloroflexi was significantly positively associated with the TN (*p* < 0.01), and Firmicutes was significant positively associated with the M (*p* < 0.01) (Figure 6A). Correlation analysis between soil parameters and fungal communities showed that Olpidiomycota was significantly positively associated with EC (*p* < 0.05), while Basidiomycota was significant positively associated with TC (*p* < 0.05), pH (*p* < 0.05), AP (*p* < 0.05), and AK (*p* < 0.01) (Figure 6B).

### 3.4. Microbial network analysis

**Figure 7:**
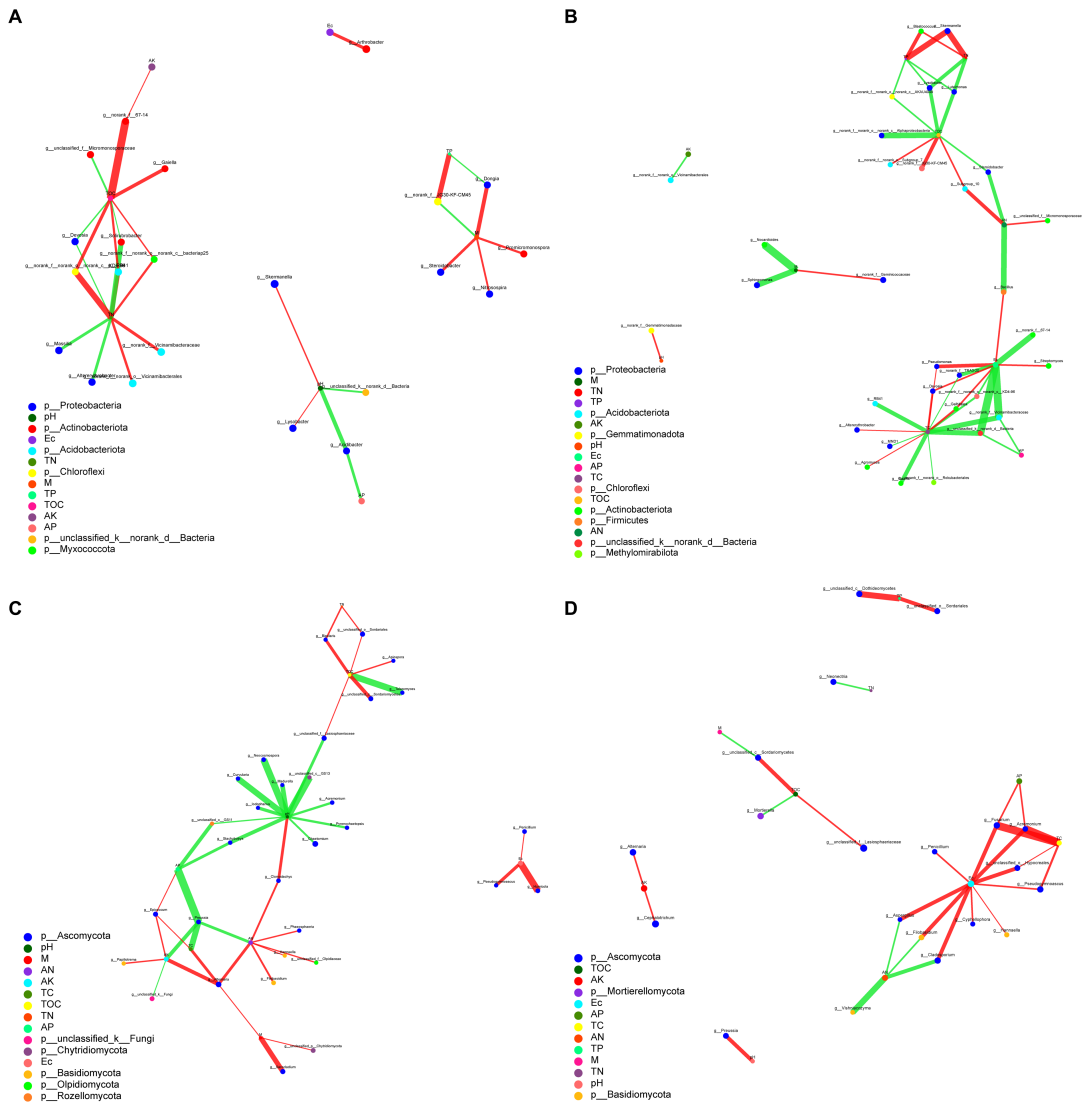
Correlation network analysis of environmental parameters and microbial communities **(A)** Biochar-bacteria. **(B)** Organic fertilizer-bacteria. **(C)** Biochar-fungi. **(D)** Organic fertilizer-fungi.

The complexity of the interactions within the microbial communities and soil parameter under biochar and organic fertilizer application was conducted by to assess their topological properties. The results showed that there was a difference between the microbial communities based on the addition of biochar and organic fertilizer (Figure 7). For bacteria, the complexity of organic fertilizer-bacteria was greater than those of biochar-bacteria, indicating that the addition of organic fertilizer increased the complexity of the correlation between bacteria and soil environmental factors (Figures 7A,B). But for fungi, the complexity of biochar-fungi was greater than those of organic fertilizer-fungi, indicating that the addition of biochar increased the complexity of the correlation between fungi and soil environmental factors. For bacteria, the average number of connections per node was higher following organic fertilizer treatment (node average degree = 2.43) than after the biochar treatment (node average degree = 2.07) (Supplementary Table S3). But for fungi, the average number of connections per node was higher following biochar treatment (node average degree = 2.25) than after the organic fertilizer treatment (node average degree = 2.00; Supplementary Table S4). Nodes with the highest connections between environmental parameters and bacteria under biochar and organic fertilizer were TN (9) and TC (13), respectively. While nodes with the highest connections between environmental parameters and fungi under biochar and organic fertilizer were pH (12) and Ec (10), respectively. This result suggests that bacteria and fungi respond differently to biochar and organic fertilizer treatments, and soil parameters have different effects on bacterial and fungal communities.

**Figure 8:**
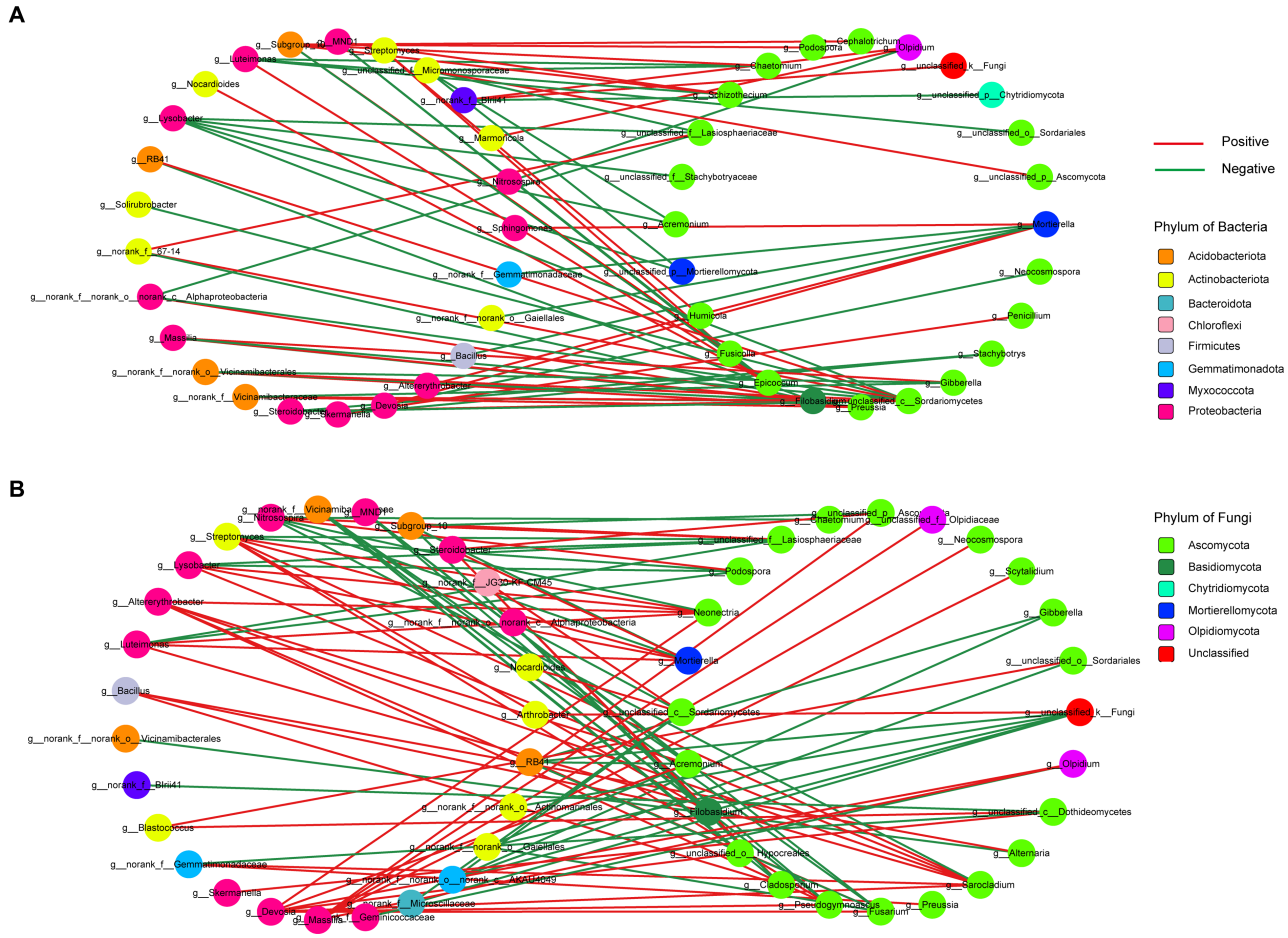
Correlation network analysis of microbial communities. Node color represents phylum classification. Edge color corre- sponds to positive (red) and negative (green) correlation, and the edge thickness is equivalent to the correlation values. **(A)** The network between biochar. **(B)** The network between organic fertilizer.

To investigate the potential interactions between bacteria and fungi in wheat soil after the application of biochar and organic fertilizer, we constructed a correlation network of microbial communities (Figure 8). Supplementary Table S5 provides an overview of several significant topological properties of this microbial community correlation network. It was observed that the number of nodes and edges in the bacterial and fungal correlation network was higher for the organic fertilizer application compared to the biochar application. Additionally, a greater number of positive correlated edges were found in the organic fertilizer application, whereas the biochar application exhibited the opposite trend. Regarding the biochar application, 91.66% of the bacterial nodes were affiliated with Proteobacteria (44%), followed by Actinobacteriota (28%) and Acidobacteriota (16%). As for the fungal nodes, 75% belonged to Ascomycota. On the other hand, for the organic fertilizer application, 77.78% of the bacterial nodes were affiliated with Proteobacteria (40.74%), Actinobacteriota accounted for 22.22%, and Acidobacteriota for 14.81%. Furthermore, 79.17% of the fungal nodes remained within the Ascomycota group.

## 4. Discussion

Although many researches have explored the effects of biochar and organic fertilizer in agricultural fields, only a few have conducted saline–alkali field experiments (Yao et al., 2017; Gu et al., 2022a, 2022b, 2023). Biochar amendment and application of organic fertilizer generally changes soil quality, such as pH, soil organic carbon, and nutrient content (Lehmann et al., 2011; Luo et al., 2014; Yao et al., 2017). In reports from Yao et al. (2017), the addition of high concentrations biochar in soil has been shown to significantly improve pH and TN. During this research, we found that the biochar and organic fertilizer caused no significant changes in pH and TN, while the TOC content showed significant change, this is like the findings of Gao et al. (2021). This was due to the high background pH of the soil (7.95) for which biochar application resulted in only a slight increase (8.05–8.07), but this result was non-statistically significant (*p* > 0.05). We found in this research that the EC value of the soil decreases with the addition of biochar and organic fertilizer. Previous study has also showed a similar decrease in soil Ec values when various organic additives were used to remediate the soil (Tejada et al., 2006). Soil AP and AK improved with the addition of biochar, which agreed with Oladele et al. (2019). This may be because some nutrients are produced by the pyrolysis of the biochar during its manufacture, and because the porous structure of the biochar causes it to adhere to more functional groups, which increases its ability to adsorb soil nutrients (Ameloot et al., 2013; Zhang et al., 2019).Organic fertilizer is known to contain nitrogen, phosphorus, and potassium. However, for this research, the addition of organic fertilizer only increased the content of AK, even with a reduced AP content. It is well known that biochar and organic fertilizers can induce variations in the soil environment, such as soil nitrogen, potassium, phosphorus levels, and pH (Sun et al., 2020). The differences between study results may be due to a variety of factors, such as the climate, soil properties, fertilizer, and plants grown (Assefa and Tadesse, 2019).Therefore, many factors need to be considered when analyzing the changes in soil properties.

It was shown that either biochar or fertilizer can change the structure of the soil microbial community and crop performance (Tao et al., 2020; Gao et al., 2021). Biochar can provide a habitat for microbiota through the provision of organic compounds, further mediating changes in microbial population structure and abundance (Gao et al., 2021), and on the other hand, toxic substances such as polycyclic aromatic hydrocarbons (PAHs) present in biochar can adversely affect soil microbial populations, which can lead to alterations in the composition of the fungal community and reductions in its diversity (Zheng et al., 2016; Sheng and Zhu, 2018). In this study, the richness of soil bacterial and fungal communities were not significantly different after biochar treatment compared to CK, but the overall trend showed an increase both in richness and diversity of soil bacterial and fungal communities with the biochar applied (Table 2), and there was a significant increase (*p* < 0.05) in diversity of soil bacterial community compared to CK under the BM, which were consistent with Yao et al. (2017) and Gao et al. (2021). Tao et al. (2020) showed that the application of organic fertilizer had a significantly higher effect on bacterial richness and diverstiy than CK, but reduced the richness and diversity of fungal species. Sun et al. (2020) also found the same fungal results as Tao et al. (2020). Chen et al. (2021) discovered that organic fertilizer application significantly enhanced the richness and diversity of fungal species, but it had no significant impact on bacteria. Our research results on bacteria were consistent with Chen et al. (2021), while fungi were consistent with Tao et al. (2020). Differences in the effects of organic fertilizer application on bacteria and fungi may be caused by differences in crops, soil conditions, types of organic fertilizer and application methods.

Effects of biochar application on the composition and structure of soil microbial communities have been frequently reported (Zheng et al., 2016; Gao et al., 2021). In this study, the predominant phyla of bacterial community in the wheat soil were Proteobacteria, Actinobacteria, Acidobacteria, Bacteroidota, and Chloroflexi, which fitted to the findings by other researchers also observed in wheat soil (Meng et al., 2019). However, the dominant fungus in this study is the Ascomycota, which is different from the Zygomycota reported by Meng et al. (2019), but consistent with Chen et al. (2021) and Zheng et al. (2016). Yao et al. (2017) found that relative abundances of Acidobacteria were decreased, while the abundances of Chloroflexi were increased with biochar addition. Khodadad et al. (2011) reported that biochar amendment decreased the bacterial diversity but increased the relative abundance of bacterial phyla Actinobacteria and Gemmatimonadetes. Our results indicated that Acidobacteria, Chloroflexi, and Gemmatimonadetes increased with biochar addition, especially BH, whereas Actinobacteria decreased. Wolna-Maruwka et al. (2021) discovered that organic fertilizer increased the abundence of Actinobacteria and Firmicute, but decreased Acidobacteria, which is partially consistent with our results that Gemmatimonadetes and Firmicutes increased with organic fertilizer addition. Acidobacteriota was significantly positively correlated with TOC (*p* < 0.05) content, it can also partially explain the significant increase in TOC of biochar application compared to CK. Both Ascomycota (copiotrophic) and Basidiomycota (oligotrophic) play important roles in plant growth by improving nutrition and defense against pests (Meng et al., 2019). Yao et al. (2017) reported that biochar addition increased the relative abundance of Ascomycota and decreased the relative abundance of Basidiomycota compared to CK. In this study, the abundance of Ascomycota increased with addition content of biochar and organic fertilizer, but this has not achieved the remarkable level. Basidiomycota, which was significantly positively correlated with the TC (*p* < 0.05), pH (*p* < 0.05), AP (*p* < 0.05), and AK (*p* < 0.01), also increased with biochar addition and decreased with organic fertilizer addition without statistically significant. This may be one of the reasons why the TC, AP, AK of biochar, especially BH, is higher than those of organic fertilizers. Gao et al. (2021) found that biochar addition significantly increased the relative abundance of Mortierellomycota when compared to CK. In contrast, in this study, whether applying biochar or organic fertilizer, Mortierellomycota significantly decreased, especially when applying organic fertilizer. Differences above may be caused by differences in environment and soil conditions, or types of organic fertilizer and biochar.

The abundance of *norank_f__JG30-KF-CM45* increased with the increase of biochar and organic fertilizer concent, and was positively correlated with TP and TOC in biochar and organic fertilizer treatments, respectively, which suggested that *norank_f__JG30-KF-CM45* may be beneficial for saline alkali land remediation. This result was consistent with Shen et al. (2022), who thought that *norank_f__JG30-KF-CM45* was beneficial genus that might potentially promote tobacco growth. Yao et al. (2022) reported that biochar addition inhibited nitrification in salt-affected irrigation-silting soil by shifting the community structures of AOB and reducing the relative abundance of dominant functional ammonia-oxidizers, such as *Nitrosospira*. In this study, the addition of biochar had no effect on the abundance of *Nitrosospira* while the addition of organic fertilizer significantly increased the abundance of *Nitrosospira*, which was consistent with the result of Lin et al. (2018). Furthermore, we also found that the relative abundance of *Bacillus* increased with biochar and organic fertilizer application rate, but only organic fertilizer application reached significant level. *Bacillus* species are commercially marketed as biopesticides, biofertilizers, and soil amendments (Cao et al., 2011). So the increase abundance of *Bacillus* with biochar (Yao et al., 2017) and organic fertilizer (Tao et al., 2020) addition would be benefit for soil physical and chemical properties. Organic fertilization reduced the relative abundances of some pathogenic genera detected in our study, such as *Alternaria*, *Gibellulopsis*, and *Cladosporium*. A similar effect of organic fertilizers was reported in Semenov et al. (2022) and Lu et al. (2020). In addition, organic amendments have been proposed as a strategy for the management of plant diseases caused by soil-borne pathogens, the suppressive effect is likely related to an increase of pathogen-antagonistic fungi, since the organic fertilizers may act as an alternative C source for the antagonists (Semenov et al., 2022). *Cladorrhinum*, a potential biological control agent, increased under long-term application of manure (Semenov et al., 2022), *Trichoderma*, which are known as biocontrol agents against plant pathogens and opportunistic avirulent plant symbionts, which can be parasites and antagonists of many phytopathogenic fungi, thus protecting plants from disease (Vinale et al., 2008), and *Humicola*, a potential antagonists for the biological control of plant diseases (Ko et al., 2011), all of above genera increased with biochar and organic fertilizer application in our study. Sordariomycetes, which was positive correlation with TOC under biochar and organic fertilizer application in this study, also increased with biochar and organic fertilizer application, and this result was consistent with Ding et al. (2017) and Yu et al. (2018). In a word, biochar and organic fertilizer application in our study had led to an increase in some beneficial and biological control bacteria, as well as a decrease in pathogenic bacteria, which contributes to soil remediation in saline alkali soils.

As it is well known, soil microorganisms do not exist in isolation, but rather coexist and jointly construct complex ecological correlation networks, leading to different important and complex interactions, including but not limited to competition, commensalism, and mutualism (Duan et al., 2021). Therefore, a comprehensive understanding of the interactions between bacteria and fungi is also crucial for improving soil system services (Zhu et al., 2022). In this study, we found that the interactions among organic fertilizer treatments formed more connections than that of biochar treatments, in the meantime, both of the content of TOC and TC were higher under biochar application compared to the organic fertilizer application, which was consistent with De Menezes et al. (2017), who thought fungi-bacteria correlations were stronger in low soil organic matter soils. Highly connected taxonomic groups in the co-occurrence network typically significantly influence microbial community structure and function irrespective of their abundance across time and space (Banerjee et al., 2018). In our study, the nodes with the highest connections were *Sarocladium*, *Devosia*, and *Streptomyces* in organic fertilizer application, with a degree of 7, and *Unclassified_c__Sordariomycetes* in biochar application, with a degree of 9, indicating that these genera were relatively active in the interaction network between bacteria and fungi under biochar and organic fertilizers application, and whether they have an impact on soil physicochemical properties and plant growth deserves further study.

## 5. Conclusion

This study provides evidence that the application of biochar and organic fertilizer has different effects on soil physicochemical properties and microbial community structure. The application of biochar and organic fertilizer significantly reduced Ec and increased nutrient content of saline-alkali soils of the YRD. pH and TP were crucial contributors in regulating the bacterial and fungal community distribution. *Sphingomonas, norank_f__Geminicoccaceae*, and *Skermanella* were the dominant bacteria genera in biochar application, while *Arthrobacter* and *Sphingomonas* were the most abundant bacteria genera in organic fertilizer application. Whether biochar or organic fertilizer application, *Chaetomium* and *Gibberella* were the most dominant fungal genera. Microbial network analysis showed that bacterial and fungal communities responded differently to biochar and organic fertilizer treatments. Compare to biochar treatments, organic fertilizer treatment increasing the complexity of bacterial communities and decreasing the complexity of fungal communities. The results of the current study provides evidence that biochar and organic fertilizer treatments affect microbial communities differently and provides new insights to remediation of saline-alkali land of the YRD. The combined application of biochar and organic fertilizer and its long-term effects need further study.

## Data availability statement

The datasets presented in this study can be found in online repositories. The names of the repository/repositories and accession number(s) can be found in the article/Supplementary material.

## Author contributions

ML contributed to the conception of the study and wrote the manuscript. YG and XG also contributed to the conception of the study. CC, HZ, and JL contributed to field investigation and sample acquisition. ZW, XL, and KY performed the experiments and data analyses. NS contributed to the editing and revising of the manuscript. All authors contributed to the article and approved the submitted version.

## Footnotes

^1^www.majorbio.com

## References

[ref1] Ameloot N. Graber E. R. Verheijen F. G. De Neve S. (2013). Interactions between biochar stability and soil organisms: review and research needs. Eur. J. Soil Sci. 64, 379–390. doi: 10.1111/ejss.12064

[ref2] Assefa S. Tadesse S. (2019). The principal role of organic fertilizer on soil properties and agricultural productivity-a review. Agric. Res. Technol. Open Access J. 22:556192. doi: 10.19080/ARTOAJ.2019.22.556192

[ref3] Banerjee S. Schlaeppi K. van der Heijden M. G. A. (2018). Keystone taxa as drivers of microbiome structure and functioning. Nat. Rev. Microbiol. 16, 567–576. doi: 10.1038/s41579-018-0024-1, PMID: 29789680

[ref4] Cao Y. Zhang Z. Ling N. Yuan Y. Zheng X. Shen B.. (2011). *Bacillus subtilis* SQR 9 can control fusarium wilt in cucumber by colonizing plant roots. Biol. Fertil. Soils 47, 495–506. doi: 10.1007/s00374-011-0556-2

[ref5] Chen J. Liu X. Zheng J. Zhang B. Lu H. Chi Z.. (2013). Biochar soil amendment increased bacterial but decreased fungal gene abundance with shifts in community structure in a slightly acid rice paddy from Southwest China. Appl. Soil Ecol. 71, 33–44. doi: 10.1016/j.apsoil.2013.05.003

[ref6] Chen H. Zhao J. Jiang J. Zhao Z. Guan Z. Chen S.. (2021). Effects of inorganic, organic and bio-organic fertilizer on growth, rhizosphere soil microflora and soil function sustainability in Chrysanthemum monoculture. Agriculture 11:1214. doi: 10.3390/agriculture11121214

[ref7] Compant S. Clément C. Sessitsch A. (2010). Plant growth-promoting bacteria in the rhizo- and endosphere of plants: their role, colonization, mechanisms involved and prospects for utilization. Soil Biol. Biochem. 42, 669–678. doi: 10.1016/j.soilbio.2009.11.024

[ref8] Dangi S. Gao S. Duan Y. Wang D. (2019). Soil microbial community structure affected by biochar and fertilizer sources. Appl. Soil Ecol. 150:103452. doi: 10.1016/j.apsoil.2019.103452

[ref9] De Menezes A. B. Richardson A. E. Thrall P. H. (2017). Linking fungal–bacterial co-occurrences to soil ecosystem function. Curr. Opin. Microbiol. 37, 135–141. doi: 10.1016/j.mib.2017.06.006, PMID: 28692866

[ref10] Demelash N. Bayu W. Tesfaye S. Ziadat F. Sommer R. (2014). Current and residual effects of compost and inorganic fertilizer on wheat and soil chemical properties. Nutr. Cycl. Agroecosyst. 100, 357–367. doi: 10.1007/s10705-014-9654-5

[ref11] Ding J. Jiang X. Guan D. Zhao B. Ma M. Zhou B.. (2017). Influence of inorganic fertilizer and organic manure application on fungal communities in a long-term field experiment of Chinese Mollisols. Appl. Soil Ecol. 111, 114–122. doi: 10.1016/j.apsoil.2016.12.003

[ref12] Duan Y. Lian J. Wang L. Wang X. Luo Y. Wang W.. (2021). Variation in soil microbial communities along an elevational gradient in alpine meadows of the Qilian Mountains, China. Front. Microbiol. 12:684386. doi: 10.3389/fmicb.2021.684386, PMID: 34248904 PMC8270674

[ref13] Fang H. Liu G. Kearney M. (2005). Georelational analysis of soil type, soil Salt content, landform, and land use in the Yellow River Delta, China. Environ. Manag. 35, 72–83. doi: 10.1007/s00267-004-3066-2, PMID: 15984065

[ref14] Gao W. Gao K. Guo Z. Liu Y. Jiang L. Liu C.. (2021). Different responses of soil bacterial and fungal communities to 3 years of biochar amendment in an alkaline soybean soil. Front. Microbiol. 12:630418. doi: 10.3389/fmicb.2021.630418, PMID: 34122356 PMC8187762

[ref15] Ge G. Li Z. Fan F. Chu G. Hou Z. Liang Y. (2009). Soil biological activity and their seasonal variations in response to long-term application of organic and inorganic fertilizers. Plant Soil 326, 31–44. doi: 10.1007/s11104-009-0186-8

[ref16] Gu Y. Y. Liang X. Y. Zhang H. Y. Fu R. Li M. Chen C. J. (2023). Effect of biochar and bioorganic fertilizer on the microbial diversity in the rhizosphere soil of *Sesbania cannabina* in saline-alkaline soil. Front. Microbiol. 14:1190716. doi: 10.3389/fmicb.2023.1190716, PMID: 37455751 PMC10339320

[ref17] Gu Y. Y. Zhang H. Y. Liang X. Y. Fu R. Li M. Chen C. J. (2022a). Impact of biochar and bioorganic fertilizer on rhizosphere Bacteria in saline-alkali soil. Microorganisms 10:2310. doi: 10.3390/microorganisms10122310, PMID: 36557563 PMC9785793

[ref18] Gu Y. Y. Zhang H. Y. Liang X. Y. Fu R. Li M. Chen C. J. (2022b). Effect of different biochar particle sizes together with bio-organic fertilizer on rhizosphere soil micro-ecological environment on saline-alkali land. Front. Environ. Sci. 10:949190. doi: 10.3389/fenvs.2022.949190

[ref19] Insam H. Gómez-Brandón M. Ascher J. (2015). Manure-based biogas fermentation residues – friend or foe of soil fertility? Soil Biol. Biochem. 84, 1–14. doi: 10.1016/j.soilbio.2015.02.006, PMID: 37601357

[ref20] Kang S. W. Kim S. H. Park J. H. Seo D. C. Ok Y. S. Cho J. S. (2018). Effect of biochar derived from barley straw on soil physicochemical properties, crop growth, and nitrous oxide emission in an upland field in South Korea. Environ. Sci. Pollut. R. 25, 25813–25821. doi: 10.1007/s11356-018-1888-3, PMID: 29654461

[ref21] Khodadad C. L. M. Zimmerman A. R. Green S. J. Uthandi S. Foster J. S. (2011). Taxa-specific changes in soil microbial community composition induced by pyrogenic carbon amendments. Soil Biol. Biochem. 43, 385–392. doi: 10.1016/j.soilbio.2010.11.005

[ref22] Ko W. H. Yang C. H. Lin M. J. Chen C. Y. Tsou Y. J. (2011). Humicola phialophoroides sp. nov. from soil with potential for biological control of plant diseases. Bot. Stud. 52, 197–202.

[ref23] Le H. T. Ho C. T. Trinh Q. H. Trinh D. A. Luu M. T. N. Tran H. S.. (2016). Responses of aquatic Bacteria to terrestrial runoff: effects on community structure and key taxonomic groups. Front. Microbiol. 7:889. doi: 10.3389/fmicb.2016.00889, PMID: 27379034 PMC4908118

[ref24] Lehmann J. Rillig M. C. Thies J. Masiello C. A. Hockaday W. C. Crowley D. (2011). Biochar effects on soil biota – a review. Soil Biol. Biochem. 43, 1812–1836. doi: 10.1016/j.soilbio.2011.04.022, PMID: 37329992

[ref25] Li H. Wang J. Liu Q. Zhou Z. Chen F. Xiang D. (2019). Effects of consecutive monoculture of sweet potato on soil bacterial community as determined by pyrosequencing. J. Basic Microbiol. 59, 181–191. doi: 10.1002/jobm.201800304, PMID: 30288775

[ref26] Lin Y. Ye G. Luo J. Di H. J. Liu D. Fan J.. (2018). Nitrosospira cluster 8a play a predominant role in the nitrification process of a subtropical Ultisol under long-term inorganic and organic fertilization. Appl. Environ. Microbiol. 84:AEM.01031–18. doi: 10.1128/aem.01031-18 PMC6121968 30006397

[ref27] Liu S. Meng J. Jiang L. Yang X. Lan Y. Cheng X.. (2017). Rice husk biochar impacts soil phosphorous availability, phosphatase activities and bacterial community characteristics in three different soil types. Appl. Soil Ecol. 116, 12–22. doi: 10.1016/j.apsoil.2017.03.020

[ref28] Liu J. Shu A. Song W. Shi W. Li M. Zhang W.. (2021). Long-term organic fertilizer substitution increases rice yield by improving soil properties and regulating soil bacteria. Geoderma 404:115287. doi: 10.1016/j.geoderma.2021.115287

[ref29] Lu P. Yang T. Li L. Zhao B. Liu J. (2020). Response of oat morphologies, root exudates, and rhizosphere fungal communities to amendments in a saline-alkaline environment. PLoS One 15:e0243301. doi: 10.1371/journal.pone.0243301, PMID: 33270753 PMC7714365

[ref30] Luo P. Han X. Wang Y. Han M. Shi H. Liu N.. (2014). Influence of long-term fertilization on soil microbial biomass, dehydrogenase activity, and bacterial and fungal community structure in a brown soil of Northeast China. Ann. Microbiol. 65, 533–542. doi: 10.1007/s13213-014-0889-9, PMID: 25705148 PMC4331610

[ref31] Luo X. Liu G. Xia Y. Chen L. Jiang Z. Zheng H.. (2016). Use of biochar-compost to improve properties and productivity of the degraded coastal soil in the Yellow River Delta, China. J. Soils Sediments 17, 780–789. doi: 10.1007/s11368-016-1361-1

[ref32] Meng L. Sun T. Li M. Saleem M. Zhang Q. Wang C. (2019). Soil-applied biochar increases microbial diversity and wheat plant performance under herbicide fomesafen stress. Ecotox Environ. Safe. 171, 75–83. doi: 10.1016/j.ecoenv.2018.12.065, PMID: 30597319

[ref33] Oladele S. Adeyemo A. Awodun M. (2019). Influence of rice husk biochar and inorganic fertilizer on soil nutrients availability and rain-fed rice yield in two contrasting soils. Geoderma 336, 1–11. doi: 10.1016/j.geoderma.2018.08.025

[ref34] Plaza C. Hernández D. García-Gil J. C. Polo A. (2004). Microbial activity in pig slurry-amended soils under semiarid conditions. Soil Biol. Biochem. 36, 1577–1585. doi: 10.1016/j.soilbio.2004.07.017

[ref35] Salas-González I. Reyt G. Flis P. Custódio V. Gopaulchan D. Bakhoum N.. (2020). Coordination between microbiota and root endodermis supports plant mineral nutrient homeostasis. Science 371:eabd0695. doi: 10.1126/science.abd0695, PMID: 33214288

[ref36] Semenov M. V. Krasnov G. S. Semenov V. M. van Bruggen A. (2022). Mineral and organic fertilizers distinctly affect fungal communities in the crop rhizosphere. J. Fungi. 8:251. doi: 10.3390/jof8030251, PMID: 35330253 PMC8949291

[ref37] Shen M. C. Zhang Y. Z. Bo G. D. Yang B. Wang P. Ding Z. Y.. (2022). Microbial responses to the reduction of chemical fertilizers in the rhizosphere soil of flue- cured tobacco. Front. Bioeng. Biotechnol. 9:812316. doi: 10.3389/fbioe.2021.812316, PMID: 35087808 PMC8787768

[ref38] Sheng Y. Zhu L. (2018). Biochar alters microbial community and carbon sequestration potential across different soil pH. Sci. Total Environ. 622-623, 1391–1399. doi: 10.1016/j.scitotenv.2017.11.337, PMID: 29890604

[ref39] Sun R. B. Chen Y. Han W. X. Dong W. X. Zhang Y. M. Hu C. S.. (2020). Different contribution of species sorting and exogenous species immigration from manure to soil fungal diversity and community assemblage under long-term fertilization. Soil Biol. Biochem. 151:108049. doi: 10.1016/j.soilbio.2020.108049

[ref40] Sun D. Meng J. Chen W. (2013). Effects of abiotic components induced by biochar on microbial communities. Acta Agric. Scandinavica Sec. B Soil Plant Sci. 63, 633–641. doi: 10.1080/09064710.2013.838991

[ref41] Tao C. Li R. Xiong W. Shen Z. Liu S. Wang B.. (2020). Bio-organic fertilizers stimulate indigenous soil Pseudomonas populations to enhance plant disease suppression. Microbiome 8:137. doi: 10.1186/s40168-020-00892-z, PMID: 32962766 PMC7510105

[ref42] Tejada M. Garcia C. Gonzalez J. Hernandez M. (2006). Use of organic amendment as a strategy for saline soil remediation: infuence on the physical, chemical and biological properties of soil. Soil Biol. Biochem. 38, 1413–1421. doi: 10.1016/j.soilbio.2005.10.017

[ref43] Vinale F. Sivasithamparam K. Ghisalberti E. L. Marra R. Woo S. L. Lorito M. (2008). Trichoderma–plant–pathogen interactions. Soil Biol. Biochem. 40, 1–10. doi: 10.1016/j.soilbio.2007.07.002, PMID: 36719232

[ref44] Wang F. Wang X. Song N. (2021). Polyethylene microplastics increase cadmium uptake in lettuce (*Lactuca sativa* L.) by altering the soil microenvironment. Sci. Total Environ. 784:147133. doi: 10.1016/j.scitotenv.2021.147133, PMID: 33895518

[ref45] Wolna-Maruwka A. Piechota T. Niewiadomska A. Kamiński A. Kayzer D. Grzyb A.. (2021). The effect of biochar-based organic amendments on the structure of soil bacterial community and yield of maize (*Zea mays* L.). Agronomy 11:1286. doi: 10.3390/agronomy11071286

[ref46] Xu F. Liu Y. Du W. Li C. Xu M. Xie T.. (2021). Response of soil bacterial communities, antibiotic residuals, and crop yields to organic fertilizer substitution in North China under wheat–maize rotation. Sci. Total Environ. 785:147248. doi: 10.1016/j.scitotenv.2021.147248

[ref47] Yao R. J. Li H. Q. Yang J. S. Wang X. P. Xie W. P. Zhang X. (2022). Biochar addition inhibits nitrification by shifting community structure of Ammonia-oxidizing microorganisms in Salt-affected irrigation-silting soil. Microorganisms 10:436. doi: 10.3390/microorganisms10020436, PMID: 35208890 PMC8878283

[ref48] Yao Q. Liu J. Yu Z. Li Y. Jin J. Liu X.. (2017). Changes of bacterial community compositions after three years of biochar application in a black soil of Northeast China. Appl. Soil Ecol. 113, 11–21. doi: 10.1016/j.apsoil.2017.01.007

[ref49] You X. Yin S. Suo F. Xu Z. Chu D. Kong Q.. (2021). Biochar and fertilizer improved the growth and quality of the ice plant (*Mesembryanthemum crystallinum* L.) shoots in a coastal soil of Yellow River Delta, China. Sci. Total Environ. 775:144893. doi: 10.1016/j.scitotenv.2020.144893, PMID: 33618299

[ref50] Yu Z. Chen L. Pan S. Li Y. Kuzyakov Y. Xu J.. (2018). Feedstock determines biochar-induced soil priming effects by stimulating the activity of specific microorganisms. Eur. J. Soil Sci. 69, 521–534. doi: 10.1111/ejss.12542

[ref51] Zhang L. Jing Y. Xiang Y. Zhang R. Lu H. (2018). Responses of soil microbial community structure changes and activities to biochar addition: a meta-analysis. Sci. Total Environ. 643, 926–935. doi: 10.1016/j.scitotenv.2018.06.231, PMID: 29960229

[ref52] Zhang J. Zhou S. Sun H. Lü F. He P. (2019). Three-year rice grain yield responses to coastal mudflat soil properties amended with straw biochar. J. Environ. Manag. 239, 23–29. doi: 10.1016/j.jenvman.2019.03.022, PMID: 30877970

[ref53] Zhao J. Ni T. Li J. Lu Q. Fang Z. Huang Q.. (2016). Effects of organic–inorganic compound fertilizer with reduced chemical fertilizer application on crop yields, soil biological activity and bacterial community structure in a rice–wheat cropping system. Appl. Soil Ecol. 99, 1–12. doi: 10.1016/j.apsoil.2015.11.006

[ref54] Zhao R. Wu J. Jiang C. Liu F. (2020). Effects of biochar particle size and concomitant nitrogen fertilization on soil microbial community structure during the maize seedling stage. Environ. Sci. Pollut. R. 27, 13095–13104. doi: 10.1007/s11356-020-07888-0, PMID: 32016861

[ref55] Zheng J. Chen J. Pan G. Liu X. Zhang X. Li L.. (2016). Biochar decreased microbial metabolic quotient and shifted community composition four years after a single incorporation in a slightly acid rice paddy from Southwest China. Sci. Total Environ. 571, 206–217. doi: 10.1016/j.scitotenv.2016.07.135, PMID: 27471985

[ref56] Zhou Z. Gao T. Van Zwieten L. Zhu Q. Yan T. Xue J.. (2019). Soil microbial community structure shifts induced by biochar and biochar-based fertilizer amendment to karst calcareous soil. Soil Sci. Soc. Am. J. 83, 398–408. doi: 10.2136/sssaj2018.08.0297

[ref57] Zhu X. Chen B. Zhu L. Xing B. (2017). Effects and mechanisms of biochar-microbe interactions in soil improvement and pollution remediation: a review. Environ. Pollut. 227, 98–115. doi: 10.1016/j.envpol.2017.04.032, PMID: 28458251

[ref58] Zhu P. Yang S. Wu Y. Ru Y. Yu X. Wang L.. (2022). Shifts in soil microbial community composition, function, and co-occurrence network of *Phragmites australis* in the Yellow River Delta. Front. Microbiol. 13:858125. doi: 10.3389/fmicb.2022.858125, PMID: 35928147 PMC9344067

