# Supplementary material for: Effects of biochar amendment and organic fertilizer on microbial communities in the rhizosphere soil of wheat in Yellow River Delta saline-alkaline soil
Source: Front Microbiol. 2023 Sep 22;14:1250453. doi: 10.3389/fmicb.2023.1250453 (PMC10556502; doi:10.3389/fmicb.2023.1250453)
Supplement: Supplementary file 1 [file Data_Sheet_1.docx]

Supplementary Material

**
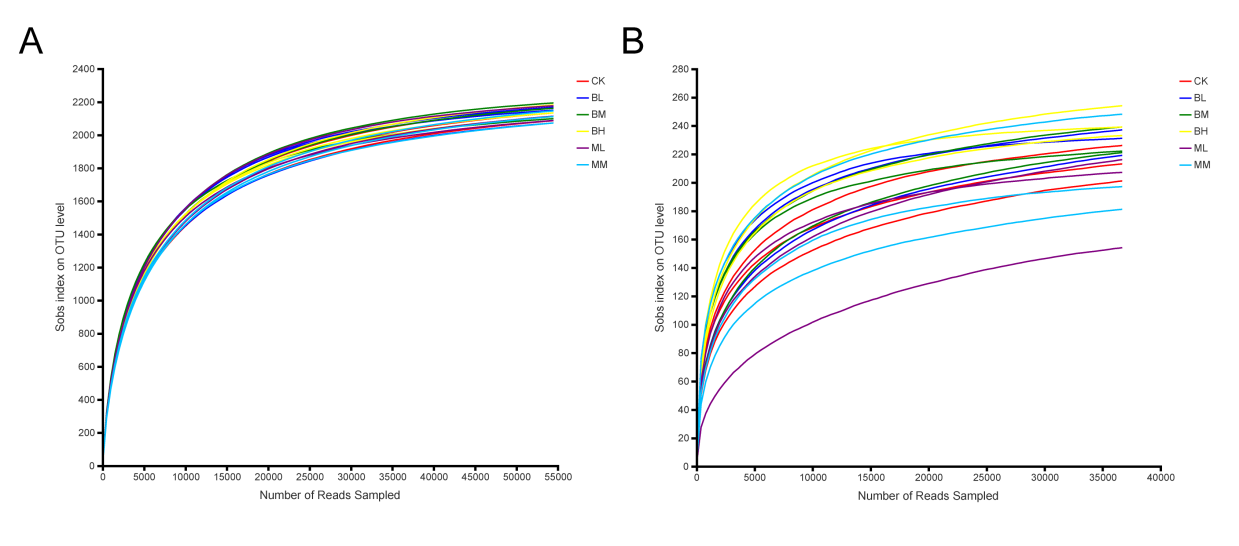
**

**Fig S1 Rarefaction curves depicting the number of (A) bacterial and (B) fungal OTUs with 97% similarity identified from different samples**

**
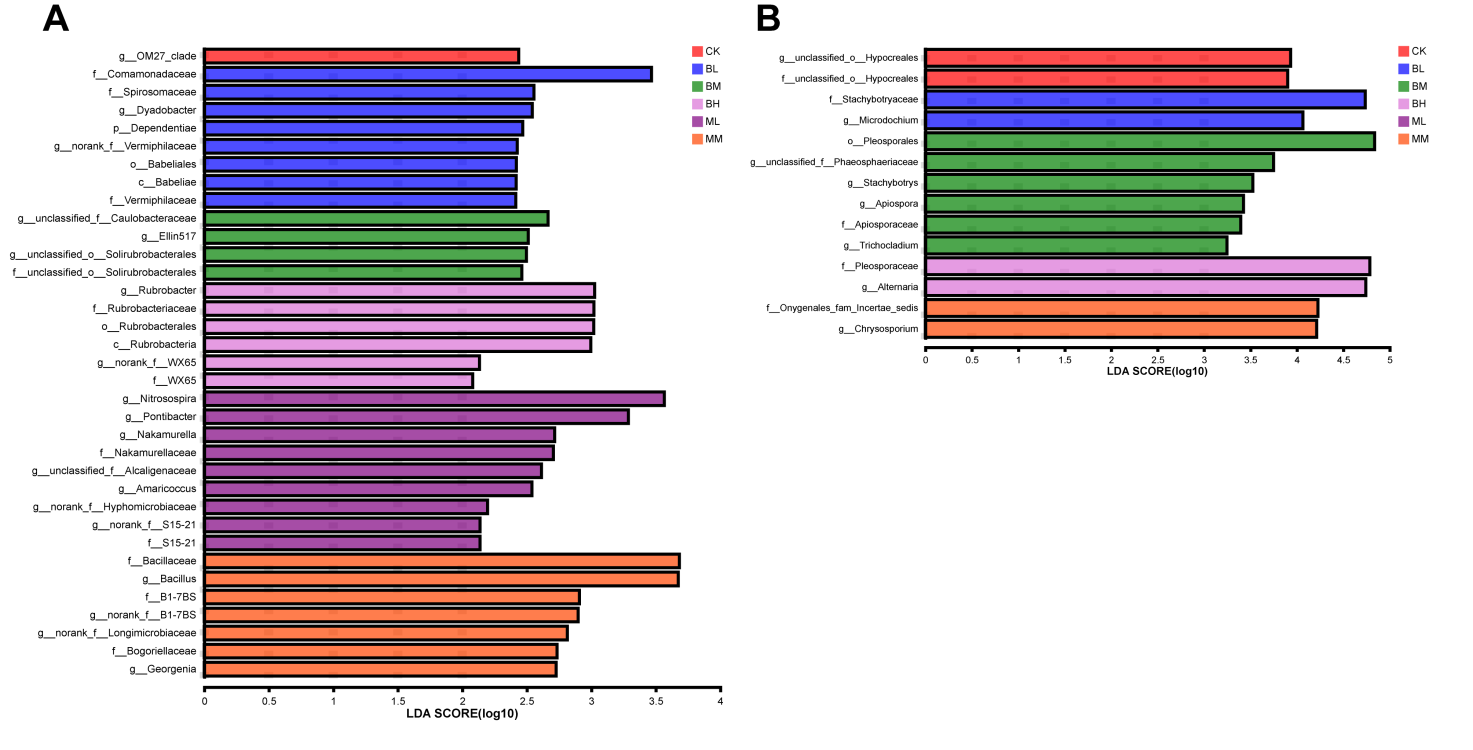
**

**Fig S2 Least discriminant analysis (LDA> 2.0) of each treated group in (A) bacterial community and (B) fungal community.**

**Table S1 Environmental explanation of the changes in soil bacterial community by RDA analysis**

|  | RDA1 | RDA2 | R^2^ | p values |
| --- | --- | --- | --- | --- |
| M | -0.29 | -0.96 | 0.14 | 0.32 |
| pH | -0.78 | 0.62 | 0.43 | 0.012 |
| EC | -0.77 | -0.64 | 0.28 | 0.077 |
| AN | 0.31 | 0.95 | 0.029 | 0.79 |
| TN | 0.99 | 0.12 | 0.35 | 0.035 |
| AP | 0.062 | 0.99 | 0.27 | 0.080 |
| TP | 0.40 | 0.91 | 0.39 | 0.017 |
| AK | 0.13 | -0.99 | 0.063 | 0.61 |
| TOC | 0.76 | 0.65 | 0.33 | 0.037 |
| TC | 0.41 | 0.91 | 0.12 | 0.39 |

**Table S2 Environmental explanation of the changes in soil fungal community by RDA analysis**

|  | RDA1 | RDA2 | R^2^ | p values |
| --- | --- | --- | --- | --- |
| M | -0.33 | 0.95 | 0.059 | 0.59 |
| pH | -0.10 | -0.99 | 0.55 | 0.002 |
| EC | 0.99 | -0.15 | 0.035 | 0.78 |
| AN | 0.82 | -0.58 | 0.035 | 0.76 |
| TN | -0.84 | 0.55 | 0.016 | 0.90 |
| AP | 0.088 | -0.99 | 0.37 | 0.016 |
| TP | 0.069 | -0.99 | 0.41 | 0.013 |
| AK | 0.97 | -0.25 | 0.11 | 0.43 |
| TOC | -0.10 | -0.99 | 0.046 | 0.69 |
| TC | 0.25 | -0.97 | 0.12 | 0.40 |

**Table S3 Correlation network analysis of bacterial communities and environment**

|  | | Biochar | Organic fertilizer |
| --- | --- | --- | --- |
| Node numbers | Soil parameter | 8 | 10 |
|  | Bacteria | 22 | 42 |
| Edge numbers | | 31 | 51 |
| Node average degree | | 2.07 | 2.43 |
| Positive edges | | 19 | 21 |
| Negative edges | | 12 | 30 |

*The correlation network indices were calculated based on the top 30 genera. The average number of connections per node in the network, that is, the node connectivity.*

**Table S4 Correlation network analysis of fungal communities and environment**

|  | | Biochar | Organic fertilizer |
| --- | --- | --- | --- |
| Node numbers | Soil parameter | 9 | 10 |
|  | Fungi | 31 | 20 |
| Edge numbers | | 45 | 30 |
| Node average degree | | 2.25 | 2.00 |
| Positive edges | | 26 | 23 |
| Negative edges | | 19 | 7 |

*The correlation network indices were calculated based on the top 30 genera. The average number of connections per node in the network, that is, the node connectivity.*

**Table S5 Correlation network analysis of microbial communities**

|  | Biochar | Organic fertilizer |
| --- | --- | --- |
| Node numbers | 49 | 51 |
| Edge numbers | 64 | 89 |
| Node average degree | 2.61 | 3.5 |
| Positive edges | 28 | 45 |
| Negative edges | 36 | 43 |

*The correlation network indices were calculated based on the top 30 genera. The average number of connections per node in the network, that is, the node connectivity.*
